# Completeness and Reliability of Location Data Collected on the Web: Assessing the Quality of Self-Reported Locations in an Internet Sample of Men Who Have Sex With Men

**DOI:** 10.2196/jmir.5701

**Published:** 2016-06-09

**Authors:** Adam S Vaughan, Michael R Kramer, Hannah LF Cooper, Eli S Rosenberg, Patrick S Sullivan

**Affiliations:** ^1^ Department of Epidemiology Rollins School of Public Health Emory University Atlanta, GA United States; ^2^ Laney Graduate School Emory University Atlanta, GA United States; ^3^ Department of Behavioral Science and Health Education Rollins School of Public Health Emory University Atlanta, GA United States

**Keywords:** HIV, digital mapping, geographic locations, survey, men who have sex with men

## Abstract

**Background:**

Place is critical to our understanding of human immunodeficiency virus (HIV) infections among men who have sex with men (MSM) in the United States. However, within the scientific literature, place is almost always represented by residential location, suggesting a fundamental assumption of equivalency between neighborhood of residence, place of risk, and place of prevention. However, the locations of behaviors among MSM show significant spatial variation, and theory has posited the importance of nonresidential contextual exposures. This focus on residential locations has been at least partially necessitated by the difficulties in collecting detailed geolocated data required to explore nonresidential locations.

**Objective:**

Using a Web-based map tool to collect locations, which may be relevant to the daily lives and health behaviors of MSM, this study examines the completeness and reliability of the collected data.

**Methods:**

MSM were recruited on the Web and completed a Web-based survey. Within this survey, men used a map tool embedded within a question to indicate their homes and multiple nonresidential locations, including those representing work, sex, socialization, physician, and others. We assessed data quality by examining data completeness and reliability. We used logistic regression to identify demographic, contextual, and location-specific predictors of answering all eligible map questions and answering specific map questions. We assessed data reliability by comparing selected locations with other participant-reported data.

**Results:**

Of 247 men completing the survey, 167 (67.6%) answered the entire set of eligible map questions. Most participants (>80%) answered specific map questions, with sex locations being the least reported (80.6%). Participants with no college education were less likely than those with a college education to answer all map questions (prevalence ratio, 0.4; 95% CI, 0.2-0.8). Participants who reported sex at their partner’s home were less likely to indicate the location of that sex (prevalence ratio, 0.8; 95% CI, 0.7-1.0). Overall, 83% of participants placed their home’s location within the boundaries of their reported residential ZIP code. Of locations having a specific text description, the median distance between the participant-selected location and the location determined using the specific text description was 0.29 miles (25th and 75th percentiles, 0.06-0.88).

**Conclusions:**

Using this Web-based map tool, this Web-based sample of MSM was generally willing and able to provide accurate data regarding both home and nonresidential locations. This tool provides a mechanism to collect data that can be used in more nuanced studies of place and sexual risk and preventive behaviors of MSM.

## Introduction

Place, or the context simultaneously experienced and defined by individuals [1], is critical to our understanding of human immunodeficiency virus (HIV) among men who have sex with men (MSM) in the United States. Through surveillance data, place fundamentally shapes our understanding of the epidemiology of the epidemic [2]. As a contextual exposure, place represents both a foundational environment in which HIV-related behaviors occur and a potential modifier of the pathway between other contextual exposures and HIV-related outcomes [3,4]. However, within the public health literature, place is almost always defined as a residential location [5-13], suggesting a fundamental assumption of equivalency between place of residence, place of sexual risk, and place of prevention. US national HIV case surveillance data make the same assumption, most often reporting data based on residence at the time of diagnosis [2].

Despite this implicit assumption, HIV-related sexual risk and prevention behaviors of MSM do not necessarily occur within the residential neighborhood [14-19]. Social ecologic theory acknowledges the importance of nonresidential locations (such as the broader urban environment and gay venues) in determining these behaviors [3,4,20-23]. For example, the availability of HIV testing services and venues where MSM gather may be influenced by broader social characteristics and norms. Access to these services and venues may then influence the formation of sexual networks and promote or inhibit individual-level behaviors, such as regular HIV testing and unprotected sex [3,4,23,24]. Consequently, using only residential neighborhood as a proxy for the many levels of sociocontextual factors may miss critical health-related exposures. To address this potential misclassification, the concept of activity spaces, which represent the collection of locations to which an individual has been exposed, has recently been introduced into the HIV literature [14,15,25].

Measuring activity spaces requires collecting large amounts of detailed geographic data. Prior studies have used global positioning systems (GPS) [26-31] or interviewer-assisted means to establish specific locations and, ultimately, to measure activity spaces [14,15]. Although these methods provide a precise and comprehensive set of locations, they have limitations. Collecting locations with GPS requires processing large amounts of data and a large investment in purchasing and maintaining the GPS devices. Interviewer-assisted methods require a large time and budget commitment, limiting the number of potential participants in a study.

To begin to address these limitations, our research group recently developed a Web-based tool that allows participants to select locations using a Google Maps question embedded within a Web-based survey [32]. Given the potentially sensitive nature of these data, participants may be more comfortable reporting such data in an anonymous Web-based survey [33]. In validation of this Web-based tool using home and health care provider locations among a cohort of HIV-positive Atlanta-area MSM, approximately 84% of participants indicated these locations using the map-based tool [32]. Among participants recruited on the Web, 50% of locations entered using the map-based tool were found to be within 0.3 miles of the true location (interquartile range, 0.1-1.1 miles). However, this previous study collected data for a limited number of locations from a population defined by a single geographic area (Atlanta, Georgia) and health status (HIV positive). Because research participation may differ by demographic and health-related factors, these results may not be generalizable to a broader population of MSM [34-37].

Therefore, given the need to gather detailed spatial data for HIV-related behaviors among MSM, to overcome current challenges in its collection, and to expand on prior validation efforts, this study examines the quality of spatial data collected using a Web-based map tool. Specifically, using a Web-based map tool to collect both residential and relevant nonresidential locations (eg, sex locations, HIV testing, work, socialization), this study examines the completeness and reliability of data collected from MSM living in a wide range of geographic locations and independent of HIV status.

## Methods

### Recruitment

Participants were recruited using Facebook banner ads, a method that has been shown to yield samples with similar risk behaviors and demographics (excepting race) as venue-based methods of recruiting MSM [37]. Ads were targeted to users based on geography and interests. A $3 donation to a charity the participant selected from a predefined list was provided as incentive.

Eligible participants were required to be male at birth, aged 18 years or older, be able to read and write English, and had to report at least one male sex partner in the past 6 months and to reside in Georgia, Texas, or Wisconsin. These 3 states vary in their underlying HIV epidemiology, demographics, and contextual factors, which could be associated with willingness to answer our map questions and allowed us to draw conclusions based on a diverse convenience sample of MSM. This population also expands on the population used in the prior validation of this tool [32]. Participants who met eligibility criteria completed a Web-based consent form.

### Collection of Place-Based Data

Consenting participants completed a Web-based survey that included demographic and behavioral questions and an item on residential ZIP code at the time of data collection.

**Figure 1:**
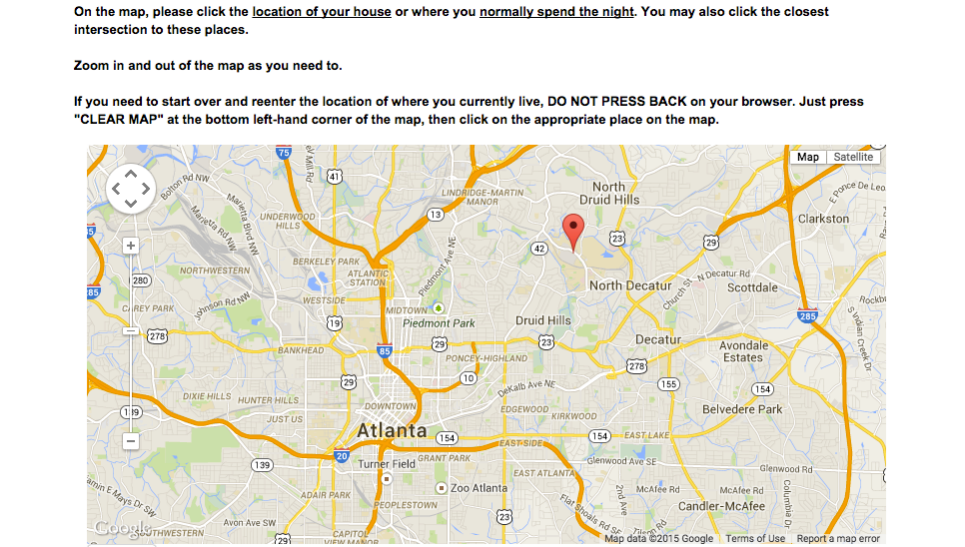
Sample of Google Maps question embedded within the Web-based survey.

In addition to these questions, participants were asked to use a map-based tool (Figure 1) [32] to drop a pin onto a Google map to indicate the following specific locations that may be relevant to the daily lives and health-related behaviors of MSM: home; work or school location, if the participant reported working at least part time or being a student; locations of up to 3 sexual encounters in the past 6 months; locations of up to 2 socialization locations; location of last HIV test, within the past year; location of the last test for another sexually transmitted infection, within the past year; primary care physician, if the participant reported having a primary care physician; pharmacy, if the participant reported having a regular pharmacy; and location where he received free condoms, if the participant reported picking up free condoms in the past 6 months.

For each location of interest, participants could choose to not answer the map question and were asked to indicate why they chose not to answer. These reasons were then categorized as either unable or unwilling to answer the question. Answer options indicating that a participant was unable to select the location were the following: “I can’t remember where this location is,” “I’m not sure where that place is on a map,” “I’m not comfortable using the map to select locations,” “This place is in a different city.” Answer options indicating that a participant was unwilling to select the location were “Didn’t feel comfortable giving that information,” “Worried about a loss of privacy,” “Worried about what friends, family, or coworkers would think.”

Participants were also allowed to indicate that a location was the same as another previously reported location (eg, report sex at home). In these cases, participants were not required to select the location a second time or to indicate a reason for not selecting the location. Willingness to use the map-based tool to answer the second location was assumed the same as that of the previously reported location.

For many types of locations, participants needed to report engaging in a qualifying behavior to be eligible to answer the corresponding map-based question. For example, participants needed to report having a regular physician before being presented with the map to identify physician location. As a result, the number of participants eligible to answer each location question varied.

In addition, for each location, participants entered a name that was used to reference that location throughout the survey. This name was entered by participants and could be generic (eg, home, work, bar) or specific (eg, Dr. Smith, Walgreens).

### Primary Outcome Definitions

This analysis uses 2 different primary outcomes: answering the entire set of map questions and answering specific map questions. A participant was considered to have answered the entire set of map questions if he used the map-based tool to indicate all locations for which he was eligible to answer. More granularly, the second outcome required participants to indicate specific eligible locations (eg, home, socialization, sex) using the map-based tool.

### Covariate Definitions

The covariates of interest in this study represent demographic variables, contextual factors related to residential location, and factors specific to given location types. All these factors could potentially be associated with an individual being unwilling or unable to answer the location-based questions.

Age was categorized into 3 groups with breaks at ages 25 and 50 years, in accordance with age group definitions used in the Centers for Disease Control and Prevention reporting of HIV surveillance data [38,39]. Due to a limited number of nonwhite participants, self-reported race was categorized as white or nonwhite. Education was categorized as high school diploma or less, any college, or college degree. HIV status was self-reported. State was defined as the state where the participant reported currently living. Each participant was asked to indicate his primary mode of transportation, and this was dichotomized into primarily using a car and primarily using other, noncar transportation.

Residential poverty and residential urbanicity were defined based on the reported residential ZIP code. Poverty was defined using ZIP code tabulation areas (ZCTA) from the US Census Bureau’s 2009-2013 five-year American Community Survey estimates and categorized as low poverty (<20% poverty), high poverty (≥20% poverty), or concentrated poverty (≥40% poverty), based on federal poverty definitions [40]. Urbanicity was defined using the 2013 National Center for Health Statistics Urban-Rural Classification Scheme for Counties [41], with the 2 most rural categories combined. For each sex location, participants reported the type of location (eg, sex partner’s home). Participants also reported any condomless anal intercourse (CAI) at last sex at each reported sex location.

### Statistical Analysis

#### Overview

After calculation of descriptive statistics for the covariates of interest, this analysis had 3 parts. We first examined factors associated with answering the entire set of map questions. Second, in an item-specific analysis, we examined factors associated with answering specific map questions (eg, home, sex locations). Finally, we examined the reliability of the reported locations.

#### Response to the Entire Set of Map Questions

Data regarding answering the entire set of map questions for which participants were eligible were first summarized by the covariates of interest. In bivariate analyses, we compared completeness across the levels of each covariate using chi-square and Fisher exact tests.

We then performed multivariable analyses to examine associations between the given covariates and answering all eligible map questions. Predictive margins methods were used with logistic regression to estimate adjusted prevalence ratios (PRs) for answering all map questions [42,43]. This method permitted direct estimation of adjusted PR, rather than an estimated prevalence odds ratio. Because we expected most men to respond to these questions (ie, the outcome is not rare) [32], the prevalence odds ratio estimated using logistic regression would be larger than the true PR and, consequently, direct estimation of the PR is preferred [44]. This method also avoids statistical convergence issues that may occur when estimating PR using other methods, such as log-binomial regression [45]. This model included the following possible predictors: age, race, poverty category for the residential ZIP code, residential urbanicity, state, education, HIV status, HIV test within the past year, and primary mode of transportation.

#### Response to Specific Map Questions

Data regarding answering specific map questions (ie, locations of home, sex, socialization, workor school, last HIV test, last sexually transmitted infection test, primary care physician, pharmacy, and free condoms) were first summarized by the covariates of interest. In bivariate analyses, we compared completeness in answering each type of map questions across the levels of each covariate using chi-square and Fisher’s exact tests. Proportions of the reasons for nonresponse were calculated.

We again used predictive margins methods with logistic regression to examine associations between the covariates of interest and answering specific map questions. Nine models were created, one for each location type. Each model included the following possible predictors of prevalence of response: age, race, residential poverty, residential urbanicity, state, education, HIV status, HIV test within the past year, and primary mode of transportation. The model for sex locations also included CAI and sex at the partner’s home. The model for reporting an HIV test location was restricted to HIV-negative participants. Each participant entered up to 2 socialization locations and up to 3 sex locations. Consequently, models for these 2 types of locations accounted for within-participant correlation using marginal models with exchangeable correlation structure.

#### Data Reliability

Data reliability was assessed using 2 methods. First, agreement between a reported ZIP code and residential location was determined. Other address information was not collected in this study. To measure this agreement, each residential location identified using the map tool was geocoded to a ZCTA, the US Census Bureau’s representation of ZIP codes. Agreement between the geocoded ZCTA and the participant self-reported ZIP code was then defined as an exact match between the 2 values.

In addition, reliability was assessed using distances between the reported location and name of the reported location. In this study, we asked men to identify locations for which they may not readily know the addresses and, consequently, for which a formal validation was not possible within this study. Therefore, for each location, participants entered text to help them identify the location in additional questions about that location. Using this text and the type of location, a Google Maps search was completed around the location selected using the map tool. If this search was informative, the distance between the reported point and the actual point were recorded. If the participant-entered text was generic (eg, doctor), rather than a specific name (eg, Dr Smith), then the driving distance between the selected location and the nearest location matching that description was recorded. Distances were summarized by those matched by a generic name, those matched by a specific name, and those matched using only a geographic location.

#### Analysis Software

Data management was performed using SAS, v9.4 (SAS Institute, Cary, NC, USA). Geocoding and spatial data manipulation were completed in R, v3.2.1 (R Foundation for Statistical Computing, Vienna, Austria) [46]. Predictive margins models were performed using SAS-callable SUDAAN, v11.0.1 (Research Triangle Institute, Research Triangle Park, NC, USA).

### Ethics

This study was approved by the Emory University Institutional Review Board (protocol #IRB00074519).

## Results

### Sample Characteristics and Question Completeness

Of 105,815 men presented with the Facebook ad, 3058 men (2.9%) clicked on the ad to enter the eligibility screening. Of these, 624 men (20.4%) were eligible, of whom 341 men (11.1% of those screened, 54.6% of those eligible) consented to participate in the study. 247 men (72.4%) completed the survey and are included in this analysis. Our sample represented a wide range of ages, urbanicity, and poverty levels (Table 1). Our sample was highly educated and largely white.

**Table 1:** Sample characteristics (N=247).

Covariate		Number (%)
**Age (years)**		
	18-25	66 (26.7)
	26-50	103 (41.7)
	51 and older	78 (31.6)
**Race**		
	White	202 (81.8)
	Non-white race	45 (18.2)
Reported HIV^a^ positive		36 (14.6)
HIV^a^ test within the past year^b^		119 (56.4)
**Education**		
	High school or less	22 (8.9)
	Some college	89 (36.0)
	College degree	136 (55.1)
**State**		
	Georgia	76 (30.8)
	Texas	134 (54.3)
	Wisconsin	37 (15.0)
**Primary mode of transportation**		
	Car	227 (91.9)
	Other	20 (8.1)
**Residential poverty**		
	Low	157 (63.6)
	High	71 (28.7)
	Concentrated	19 (7.7)
**Urbanicity**		
	Urban core	108 (43.7)
	Suburban	48 (19.4)
	Medium metro	41 (16.6)
	Small metro	31 (12.6)
	Nonmetropolitan	19 (7.7)

^a^HIV: human immunodeficiency virus.

^b^Among participants who do not report being HIV positive.

### Response to the Entire Set of Map Questions

Of included participants, 167 (67.6%) answered all map questions for which they were eligible. Nine participants (3.6%) answered none of the map questions for which they were eligible. Of the remaining participants, 71 (28.7%) answered at least one, but not all, map questions.

**Figure 2:**
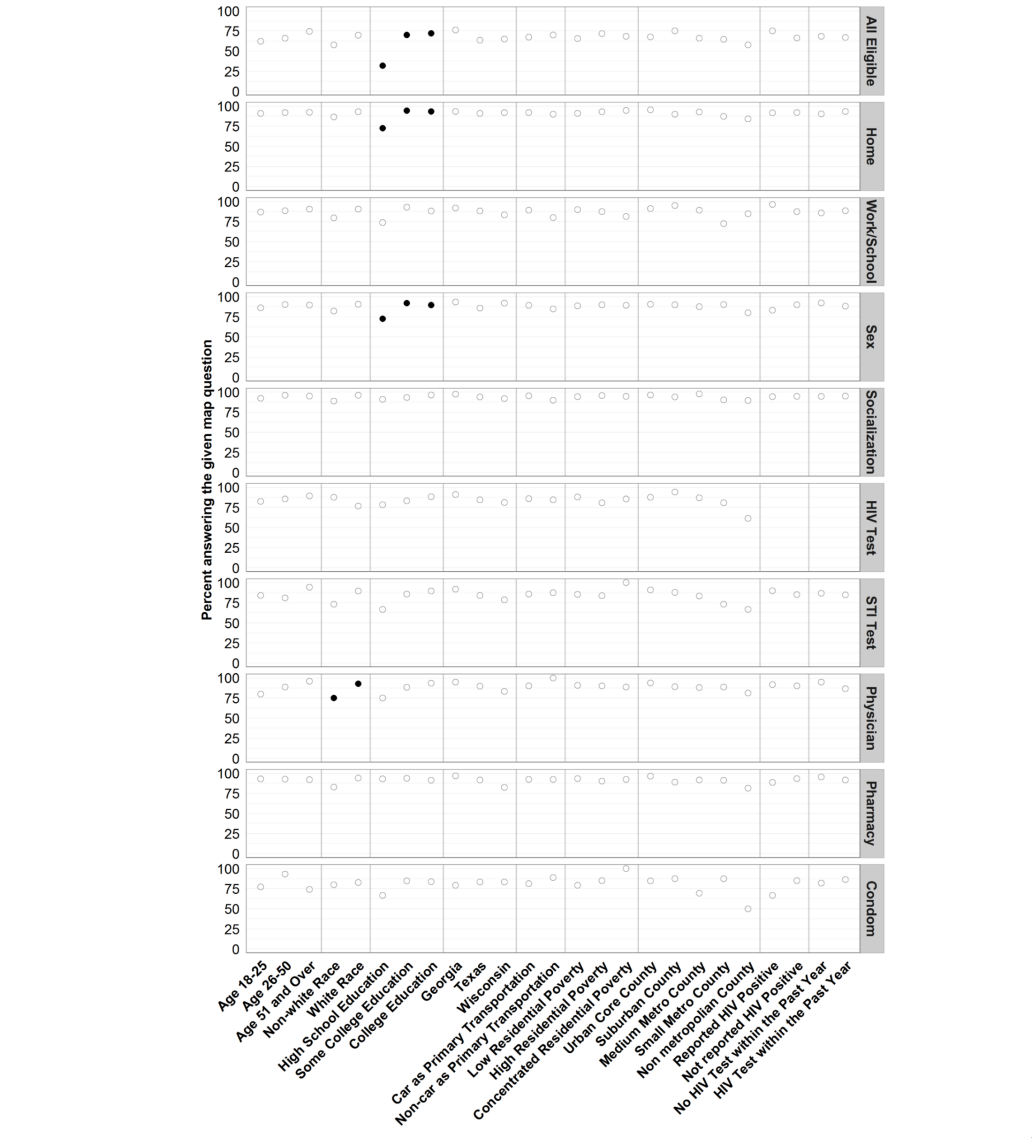
Crude percent of participants answering all eligible map questions and specific map questions. Percentages are the proportion of individuals within the given covariate level eligible to answer the map question who completed the given map question. Statistically significant differences are indicated in black filled circles.

**Figure 3:**
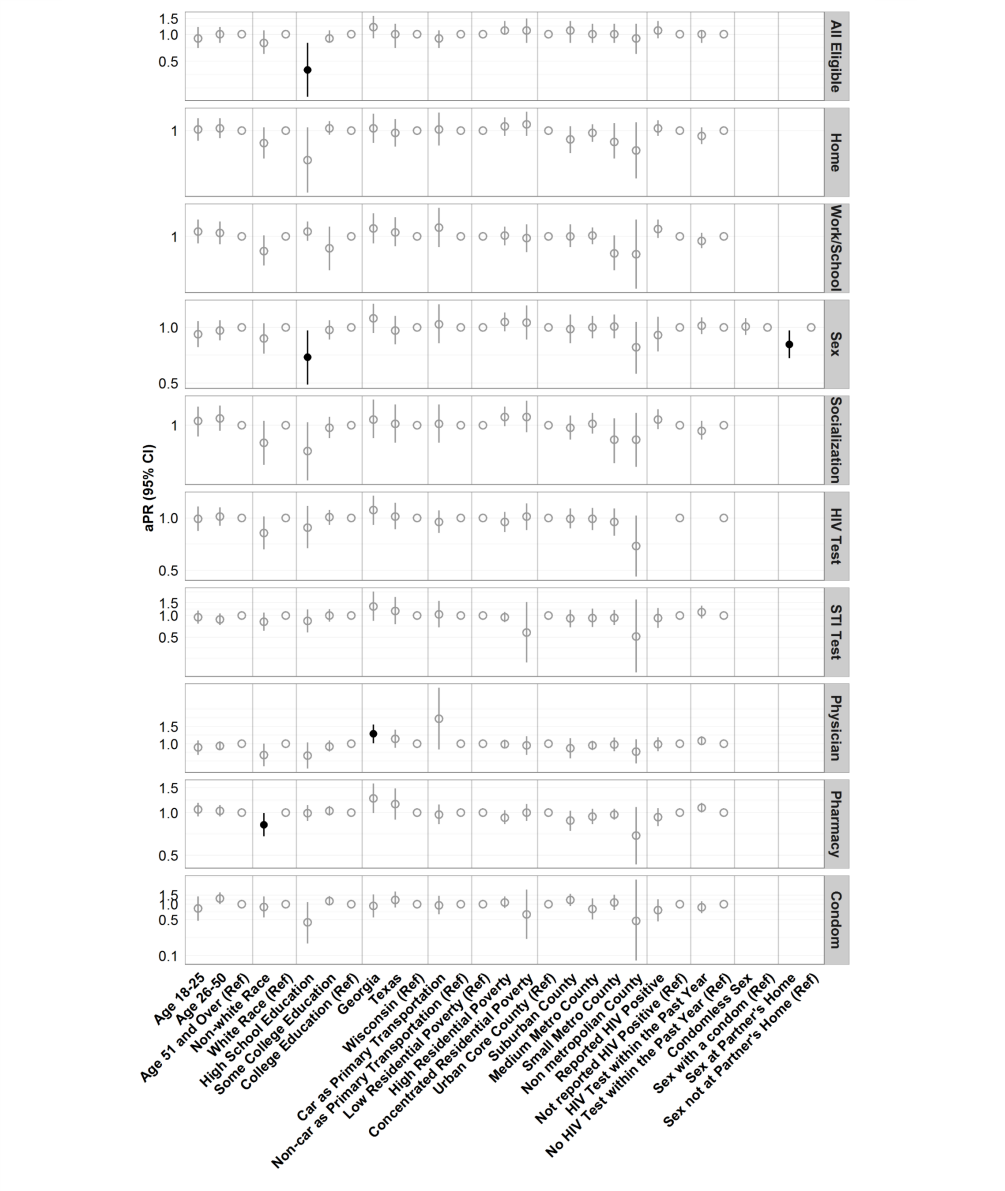
Adjusted prevalence ratios (aPR) and 95% CI for answering all eligible map questions and specific map questions by each covariate. aPRs are adjusted by all other covariates. Statistically significant aPRs are indicated with black filled circles. The scale of the y axis is logarithmic and differs across location types to better visualize the CI.

In unadjusted analyses (Figure 2), only less education was associated with significantly less completion of all map questions (*P*<.001), with 31.8% of participants with a high school diploma or less answering all questions, compared with 70.0% of participants with some college and 72.1% of participants with a college degree. This finding was confirmed in adjusted analyses (Figure 3), with participants with no college education being roughly half as likely as those with a college education to answer all eligible map questions (PR, 0.4; 95% CI, 0.2-0.8). No other covariate was significantly associated with answering all eligible map questions in unadjusted or adjusted analyses.

### Response to Specific Map Questions

In item-specific analyses, most (>80%) of those eligible answered each individual map question (Table 2). Sex locations were the least likely to be answered (80.6%). For most locations, participants who chose to not answer the map-based question were generally unwilling to answer, rather than unable to answer (Table 2). However, for sex locations and HIV testing locations, the proportion of participants who were unable to answer was similar to the proportion who were unwilling to answer.

**Table 2:** Ability and willingness to answer specific map-based questions.

Location	Total eligible (%)	Answered (%)	Unable (%)	Unwilling (%)	Both unwilling and unable (%)	No reason given (%)
Home	247 (100)	227 (91.9)	2 (0.8)	15 (6.1)	3 (1.2)	0 (0)
Work or school	209 (84.6)	185 (88.5)	2 (1.0)	21 (10.0)	0 (0)	1 (0.5)
Socialization^a^	474 (96.0)	430 (90.7)	6 (1.3)	33 (7.0)	3 (0.6)	2 (0.4)
Sex^b^	396 (53.4)	319 (80.6)	30 (7.6)	36 (9.1)	3 (0.8)	8 (2.0)
HIV^c^ test^d^	119 (56.4)	103 (86.6)	9 (7.6)	7 (5.9)	0 (0)	0 (0)
STI^c^ test	120 (48.6)	103 (85.8)	7 (5.8)	11 (11.2)	1 (1.0)	3 (3.1)
Physician	178 (72.1)	161 (90.4)	5 (2.8)	7 (13.7)	0 (0)	3 (5.9)
Pharmacy	183 (74.1)	169 (92.3)	3 (1.6)	7 (5.9)	0 (0)	0 (0)
Free condoms	78 (31.6)	64 (82.1)	3 (3.8)	9 (7.5)	1 (0.8)	0 (0)

^a^Participants reported up to 2 socialization locations.

^b^Participants reported up to 3 sex locations.

^c^HIV: human immunodeficiency virus; STI: sexually transmitted infection.

^d^Among participants who do not report being HIV positive.

In unadjusted analyses, less than college education was associated with not reporting home location (*P*=.003) and sex locations (*P*=.05) (Figure 2). Nonwhite race was significantly associated with not reporting physician (*P*=.01) locations. Sex at the partner’s house was significantly associated with not reporting the sex location (*P*=.001). No other bivariate associations were statistically significant.

In adjusted analyses, only 4 covariates were significantly associated with answering specific map questions (Figure 3). Nonwhite participants were less likely than white participants to locate a pharmacy (PR, 0.8; 95% CI, 0.7-1.0). Participants living in Georgia were more likely than participants living in Wisconsin to locate a primary care physician (PR, 1.3; 95% CI, 1.0-1.6). Participants reporting sex at their partner’s home were less likely to indicate the sex location (PR, 0.8; 95% CI, 0.7-1.0). Similarly, participants with less than a college education were less likely to indicate a sex location than participants with a college degree (PR, 0.7; 95% CI, 0.5-1.0).

No other model-based associations between the covariates and answering specific map questions were statistically significant. For example, participants who reported CAI were no more likely to report sex locations (PR, 1.0; 95% CI, 0.9-1.1).

### Data Accuracy

Of the 226 participants whose map-based home location could be assigned to a ZCTA, 187 (83%) placed the home location within the boundaries of the reported residential ZIP code. Of the 39 participants (17%) who placed a home location outside of the boundaries of the reported residential ZIP code, 29 placed the home location in an adjacent ZIP code, 2 reported post office box or institutional ZIP codes with a correct pin drop, and 8 placed the home location in a nonadjacent ZIP code. Reliability of residential location did not vary with urbanicity (*P*=.15).

Of the 1176 unique locations reported by the participants, the combination of the location type and the participant’s text description permitted 575 locations (49%) to be identified. Of these, 278 text descriptions (48%) were a specific name (eg, Walgreens), 61 (11%) were a geographic area (eg, downtown, San Antonio), and 236 (41%) were a generic name (eg, doctor, pharmacy, hospital). Of the 61 locations identified as a geographic area, 53 (87%) were placed in the correct geographic area. Locations were not able to be identified because of a name that had meaning only to the participant (eg, home, work, guy 2’s place, RLD).

Of all locations having a specific text description, the median distance between the participant-selected location and the location determined using the specific text description was 0.29 miles (interquartile range, 0.06-0.88). Of all locations having a generic text description, the median distance between the selected location and the location determined using the generic text description was 0.29 miles (IQR, 0.08-0.64). When stratified by location type, median distances between the selected location and location determined using the text descriptions were generally <one-third mile (Table 3). Although home and work have the highest median distances, very few locations could be identified based on the participant’s text description.

**Table 3:** Distance in miles between selected location and location determined using any text description.

Location	Count	Median	IQR
Home	4	0.61	0.49-0.64
Work	9	0.77	0.57-2.88
Socialize	154	0.33	0.09-0.92
Doctor	141	0.19	0.05-0.65
Pharmacy	45	0.37	0.12-0.89
Sex	86	0.34	0.10-0.74
Condoms	31	0.08	0.01-0.50
HIV^a^ test	19	0.22	0.01-0.52
STD test	24	0.22	0.13-0.49

^a^HIV: human immunodeficiency virus; STD: sexually transmitted disease.

## Discussion

### Principal Findings

In this paper, we examined the feasibility of collecting location-based data using a Web-based, map-based tool among an online convenience sample of MSM. Overall, participants were willing and able to use this tool to accurately indicate the requested locations, suggesting that this method of data collection is feasible, and results in complete, good quality data. In addition, for most locations, men who chose to not use the map tool were not significantly different from men who did use the tool with respect to demographic factors and HIV-related behaviors. The notable exception to this finding is that men were 20% less likely to report a sex location if that location was a partner’s home, reflecting both confidentiality concerns and uncertainty in the exact location.

The lack of significant associations between the examined covariates and using the map tool has critical implications for the use and subsequent interpretation of these data. Analyses relying on these locations in similar Web-based populations will have minimal bias resulting from nonresponse to these questions, with respect to the covariates measured in this study, although bias may exist due to nonparticipation. A first key exception to this finding was the observed educational gradient in which participants with no college education were less likely to provide all requested locations and sex locations. Missing data among these individuals may especially be a concern in Web-based research, where MSM of color are more difficult to recruit [47].

A second key exception is the potential for bias in analyses using sex location when sex occurs at the partner’s home (although a large majority still provided this location). Therefore, these missing data may bias analyses where either having sex at the partner’s home or education is associated with both the exposure and outcome [48]. This finding may be critical for confounding by education because lower levels of education are frequently associated with locations and with poorer health outcomes.

Men who did not provide the requested locations were generally unwilling, rather than unable, to provide the locations. Even in an anonymous Web-based survey, privacy remained a concern among a small fraction of participants. Although most participants responded to these map questions, privacy concerns for these few individuals must be considered in the implementation and interpretation of future surveys. Providing participants with the opportunity to learn more about their data’s security and reinforcing the acceptability of reporting approximate locations (eg, the nearest intersection) may help to assuage these concerns.

Similarly, participants’ inability to provide these locations could also be addressed within the Web-based survey. This inability may stem from a lack of geographic knowledge or uncertainty in locations. Incorporating text search boxes to search for a given street name or emphasizing the acceptability of identifying an intersection or other landmark could potentially address this limitation. This recommendation could also reduce the observed educational gradient in responding to these questions.

As with all participant-reported data, reliability is an important concern. Despite asking numerous locations for which participants may not readily know an address, we found good agreement between the reported locations and other reported characteristics of those locations. These results are similar to the results of a prior validation of this tool for home and treatment locations among HIV-positive MSM [32]. Participants generally placed home locations within the correct ZIP code and placed other types of locations near the probable true location. This finding suggests that, although precise measures should be used with caution, within-person and between-person relative measures are likely appropriate.

Our findings with respect to answering specific questions contrast with past studies of broader Web-based survey participation. These studies found differential participation in Web-based surveys by demographic and health-related factors. Nonurban MSM have participated in Web-based surveys more than their urban counterparts [34,35]. In addition, individuals with a given medical condition are more likely to participate in research about that condition [36], suggesting that HIV-negative men could have been less likely to provide the requested data compared with men living with HIV.

Compared with previous validation studies [32], this analysis has expanded both the population and types of locations for which valid Web-based map data may be collected. We included MSM, independent of HIV status, from urban, suburban, and rural locations, not only large urban areas that are the typical geographic focus of much HIV research. We also included a wide variety of nonresidential locations that may be contextually important to the health of MSM.

As this study verified that these nonresidential location data can be collected from online samples of MSM, these locations may now be used to describe the activity spaces of MSM and to explore associations between nonresidential places and HIV-related behaviors among MSM. This Web-based tool will permit these location data to be collected using relatively low-resource methods that preserve participants’ anonymity. The results of future analyses may allow us to better consider how differing contexts are associated with HIV risk and prevention. National surveillance data, which are based on residential locations, may be interpreted differently depending on the spatial variation in HIV-related behaviors. In addition, future analyses may permit interventions and policy to be geographically targeted using the locations of relevant behaviors, rather than residential locations.

### Limitations

Despite the breadth of data being collected, this study does have limitations. First, the generalizability of this study may be limited. Our online convenience sample is likely not representative of MSM in Texas, Wisconsin, and Georgia. Our sample is less racially diverse, younger, and more educated compared with the general populations in these states. In addition, despite the breadth of HIV epidemiology, demographic, and contextual factors represented by these states, these MSM may not be representative of MSM across the United States. However, prior studies using venue-time–based sampling of MSM reported demographics similar to this study and to the Internet samples of MSM [37,47,49].

This analysis produced fully-adjusted measures of association for a large number of outcomes and their potential predictors. Consequently, some of these measures may be statistically significant due to type 2 error.

This analysis also used participant-reported ZIP codes as the basis for poverty and urbanicity measures. The use of areas to represent contextual variables may lead to misclassification, especially when using ZCTAs to represent ZIP codes [50,51]. The degree of this misclassification may be less in more urban areas [52-55], although this was not true in our predominantly urban sample. However, ZIP codes are a geographic measure that is readily accessible to participants and are therefore useful despite their limitations.

This study also was unable to validate all locations using a physical address. With our study’s expansion to locations that include where individuals socialized and had sex, validation becomes more difficult as participants may not readily know addresses of these nonresidential locations. Consequently, data reliability could be assessed only using the methods we used. In addition, the text descriptions of these places were useful for only half of locations, limiting conclusions regarding reliability of the remaining half of locations. It is possible that the half of locations that could be validated may have favorably biased the calculated accuracy. Additional validation of geographic reliability may be the subject of future work.

### Conclusions

Using a Web-based map tool, MSM participants were generally willing and able to indicate all requested locations. Critically, although most MSM reported sex locations, these locations were reported less frequently than all other locations. Consequently, within this Web-based setting and MSM population (and with careful consideration of the potential biases associated with Web-based research in this population), this method of data collection is feasible, resulting in highly complete, good quality location data.

## Abbreviations

CAI: condomless anal intercourse
GPS: global positioning systems
HIV: human immunodeficiency virus
MSM: men who have sex with men
POR: prevalence odds ratio
PR: prevalence ratio
STI: sexually transmitted infection
ZCTA: zip code tabulation area
